# New Insights into the Alveolar Epithelium as a Driver of Acute Respiratory Distress Syndrome

**DOI:** 10.3390/biom12091273

**Published:** 2022-09-10

**Authors:** Marilia Sanches Santos Rizzo Zuttion, Sarah Kathryn Littlehale Moore, Peter Chen, Andrew Kota Beppu, Jaime Lynn Hook

**Affiliations:** 1 Women’s Guild Lung Institute, Division of Pulmonary and Critical Care Medicine, Department of Medicine, Cedars-Sinai Medical Center, Los Angeles, CA 90048, USA; 2 Lung Imaging Laboratory, Division of Pulmonary, Critical Care, and Sleep Medicine, Department of Medicine, Icahn School of Medicine at Mount Sinai, New York, NY 10029, USA; 3 Department of Biomedical Sciences, Cedars-Sinai Medical Center, Los Angeles, CA 90048, USA; 4 Regenerative Medicine Institute, Cedars-Sinai Medical Center, Los Angeles, CA 90048, USA; 5 Global Health and Emerging Pathogens Institute, Department of Microbiology, Icahn School of Medicine at Mount Sinai, New York, NY 10029, USA

**Keywords:** ARDS, alveolar epithelium, lung repair, lung regeneration, lung remodeling

## Abstract

The alveolar epithelium serves as a barrier between the body and the external environment. To maintain efficient gas exchange, the alveolar epithelium has evolved to withstand and rapidly respond to an assortment of inhaled, injury-inducing stimuli. However, alveolar damage can lead to loss of alveolar fluid barrier function and exuberant, non-resolving inflammation that manifests clinically as acute respiratory distress syndrome (ARDS). This review discusses recent discoveries related to mechanisms of alveolar homeostasis, injury, repair, and regeneration, with a contemporary emphasis on virus-induced lung injury. In addition, we address new insights into how the alveolar epithelium coordinates injury-induced lung inflammation and review maladaptive lung responses to alveolar damage that drive ARDS and pathologic lung remodeling.

## 1. Introduction

The lungs maintain a sizeable interface (equivalent to a tennis court) between the body and the external environment. This large surface area is necessary to facilitate efficient gas exchange for the body’s consumptive needs but also exposes the lung epithelial surface to potentially noxious stimuli, leading to alveolar damage and lung injury. Recovery from injury requires that the epithelium repair or regenerate to reestablish a competent barrier. In carrying out these processes, the alveolar epithelium modulates inflammatory signaling to eliminate pathogens and promote lung repair. 

In the face of overwhelming injury, however, physiologic repair and regeneration mechanisms may be insufficient to reverse the epithelial damage. In this setting, pathologic signaling pathways are activated that lead to maladaptive repair and inflammation. Initially, the exuberant, non-resolving lung inflammation manifests clinically as acute respiratory distress syndrome (ARDS). Persistence of the maladaptive signals may activate lung remodeling programs that are fibroproliferative and destructive, resulting in potentially permanent lung dysfunction. 

In this review, we will limit our discussion to the alveolar epithelium in order to provide an update on the physiologic and pathologic responses to inhaled challenge that promote alveolar injury, repair, regeneration, and remodeling, particularly in the context of ARDS. By highlighting recent work in this area, we hope to build a more complete picture of how the alveolar epithelium maintains alveolar homeostasis and contributes to ARDS development.

## 2. The Alveolar Epithelium at Homeostasis

### 2.1. Alveolar Epithelial Structure

**Figure 1:**
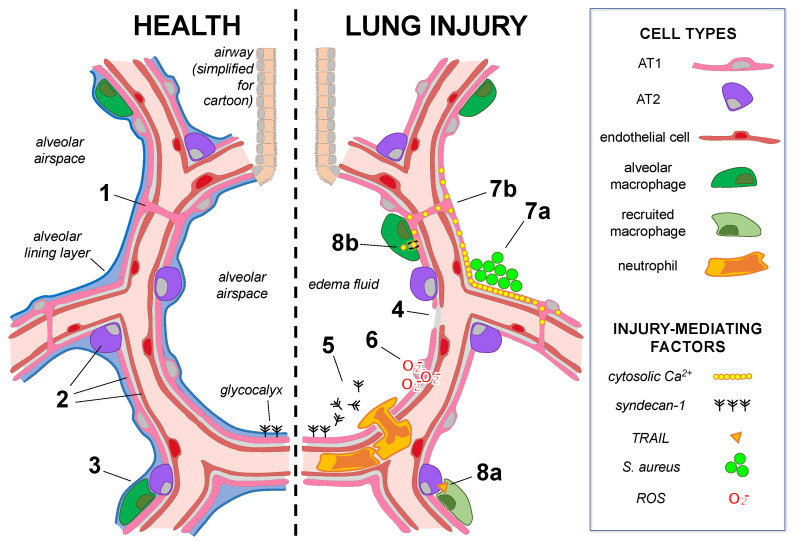
**Mechanisms of alveolar epithelial health and lung injury.** The cartoon shows alveoli under baseline conditions (**left**) in which alveolar epithelial type 1 (*AT1*) and type 2 (*AT2*) cells underlie the alveolar lining layer. The glycocalyx extends from the epithelial surface. (Note: the glycocalyx size is exaggerated.) AT1 cells span multiple alveoli through the pores of Kohn in human lungs (**1**), and AT1, AT2, and microvascular endothelial cells (**2**) are heterogeneous groups comprised of subpopulations. AT2-cell-derived GM-CSF (**3**) maintains the lung resident alveolar macrophage population in healthy adult lungs. In lung injury (**right**), alveolar epithelial cell damage, junctional and Na,K-ATPase protein loss (**4**), and surfactant dysfunction lead to edema fluid accumulation in airspaces. Here, we highlight examples of the cell-autonomous and extrinsic interactions by which the alveolar epithelium responds to injury (detailed in Section 3). (**5**) *Alveolar responses to injury*: Glycocalyx shedding promotes airspace neutrophil recruitment and activation. (**6**) *Epithelial–endothelial crosstalk*: ROS release from the injured alveolar epithelium causes mitochondrial depolarization and cytoskeletal destabilization in the epithelium-adjacent endothelium. (**7a,b**) *Alveolar epithelial cell–cell communication*: (**7a**) Inhaled *S. aureus* form microaggregates in structural alveolar niches, where localized increases in cytosolic Ca^2+^ spread (**7b**) through alveolar epithelial gap junctions to induce widespread alveolar edema. (**8a,b**) *Reciprocal signaling between the alveolar epithelium and immune cells*: (**8a**) Interferon alpha secretion by influenza-infected AT2 cells induces secretion of TNF-related apoptosis-induced ligand (TRAIL) by recruited macrophages, inducing signaling to the uninfected alveolar epithelium, which leads to epithelial loss of Na,K-ATPase protein. (**8b**) Alveolar macrophages directly interact with the alveolar epithelium via gap junctions to communicate anti-inflammatory cytosolic Ca^2+^ signals, limiting lung injury.

Structural features of the alveolar epithelium include the alveolar epithelial cell monolayer and alveolar lining layer (Figure 1). The cellular monolayer is a mosaic of squamous alveolar type 1 (AT1) and cuboidal alveolar type 2 (AT2) cells whose basement membranes abut the alveolar interstitium. Opposite the interstitium lie the basement membranes and cells of the pulmonary microvascular endothelium. Together, the alveolar epithelium, alveolar interstitium, and microvascular endothelium form the alveolar barrier.

The alveolar barrier facilitates gas exchange between air and blood compartments of the lung. As reviewed by Weibel [1], the body’s metabolic needs require that gas exchange be efficient; thus, the alveolar barrier must be thin and extensive. AT1 cells are well-suited to gas exchange owing to their attenuated cytoplasmic leaflets, which are in many places less than 0.2 µm thick and spread broadly across adjacent alveoli to span an average surface area of 5000 µm^2^ per cell in human lungs [1,2,3,4]. AT2 cells have a smaller apical surface area of approximately 200 µm^2^ per cell but are more numerous [3]. Across the mammalian spectrum, increased body mass correlates primarily with increased AT1 and AT2 cell number [5], resulting in an average alveolar epithelial surface area of more than 100 m^2^ in adult human lungs [2,6]—a remarkable size.

The alveolar lining layer is located between the apical alveolar epithelial surface and alveolar airspace. It is thin and continuous but pools at alveolar curvatures, where alveolar septa converge [7]. The lining layer consists of two phases: an aqueous hypophase in contact with the alveolar epithelium and a surfactant film that forms the air–liquid interface [8,9]. The hypophase contains reserve surfactant material [8,9] and the alveolar epithelial glycocalyx—a multilayered structure of proteoglycans and glycoproteins anchored to apical membranes of alveolar epithelial cells (reviewed in [10]).

### 2.2. Alveolar Epithelial Homeostatic Function

Homeostatic functions of the alveolar epithelium maintain the integrity of the apical lung surface and ensure that the alveolar airspaces remain air-filled for gas exchange. Maintenance of air-filled alveoli requires that the alveolar epithelium secrete surfactant and hypophase liquid and resist mechanical forces, including microvascular hydrostatic pressure, that tend to drive fluid from microvessels into airspaces (reviewed in [1]). Maintenance of apical lung surface integrity requires that the alveolar epithelium defend itself against inhaled pathogens and undergo basal cell turnover as needed.

The alveolar epithelial barrier restricts the paracellular passage of molecules, ions, and liquid into alveolar airspaces [11,12,13,14]. Epithelial barrier permeability is determined primarily by the expression and regulation of tight junctional proteins, particularly those of the claudin family [12,15,16,17]. Adherens junction proteins and ion transport-independent functions of the epithelial Na,K-ATPase contribute to barrier function by promoting epithelial and junctional protein integrity [18,19,20,21] (reviewed in [22,23,24]). Under basal conditions, therefore, junctional and Na,K-ATPase proteins separate the alveolar epithelial apical and basolateral membranes to establish epithelial polarity and regulate the paracellular movement of solutes and liquid, thereby preventing spontaneous edema formation and maintaining airspaces for gas exchange. However, when excess fluid is introduced into alveolar airspaces, apical epithelial Na^+^ channels (ENaC) and the basolateral Na,K-ATPase drive transepithelial Na^+^ uptake [25,26,27,28,29,30], generating electrochemical and osmotic gradients that induce absorption of Cl^−^ and water. The excess liquid is likely resorbed by the lymphatic system via the interstitium [31,32]. 

The alveolar lining layer is secreted by the alveolar epithelium and is essential to the gas exchange function of alveoli. Alveolar surfactant is a heterogeneous mixture of lipids and proteins that lowers surface tension at the air–liquid interface to maintain airspace patency [33,34,35] (reviewed in [36,37]). Surfactant is stored in AT2 cell lamellar bodies and secreted into the hypophase in response to lung inflation and other stimuli before it is taken up, processed, and re-secreted by AT2 cells [38] (reviewed in [39]). The hypophase provides a medium for surfactant system components and is generated continuously by chloride-dependent liquid secretion into alveolar spaces through the alveolar epithelial cystic fibrosis transmembrane conductance regulator (CFTR) protein [14,40,41,42]. 

The alveolar epithelium defends alveoli against cellular damage induced by inhaled particles and pathogens. AT2 cell secretion of granulocyte–macrophage colony-stimulating factor (GM-CSF) maintains the steady-state population of lung resident AMs [43], cells whose surveillance and phagocytosis functions are critical to alveolar defense. AT2 cell-derived surfactant proteins (SP)-A and SP-D promote AM opsonization and phagocytosis of pathogens and directly inhibit growth of Gram-negative bacteria and fungi [44,45]. Alveolar epithelial hypophase secretion may facilitate AM function by retaining and perhaps opsonizing inhaled particles [46]. In addition, hypophase secretion contributes directly to alveolar defense through pH-mediated regulation of SP-A and SP-D activity [44,47] and generation of alveolar liquid flow that convectively transports particles out of alveoli [40].

To maintain gas exchange over a lifetime, the alveolar epithelium must undergo homeostatic cell turnover. Recent research sheds new light on alveolar epithelial turnover rates and mechanisms. In mice, lineage tracing studies indicate homeostatic alveolar epithelial turnover is a slow process [48,49]. The turnover mechanism may depend on juxtacrine interactions between alveolar interstitial fibroblasts and AT2 cells [48], which serve as stem cells for the alveolar epithelium [50]. Notably, a rare AT2 cell subpopulation appears to have higher replication capacity under baseline conditions [51]. Cells of this subpopulation tend to be located near pulmonary microvessels [51], but their role in homeostasis, the significance of their location, and the extent to which the cells communicate with microvascular endothelia or pericapillary cells remains unknown. The recent finding that the alveolar capillary endothelium is also comprised of subpopulations [52] raises the intriguing possibility that specialized cells of the alveolar epithelium and adjacent microvascular endothelium interact to maintain alveolar health.

## 3. Alveolar Epithelial Responses That Lead to Lung Injury 

### 3.1. New Insights into ARDS Pathogenesis from Human Studies during the COVID-19 Pandemic

The destructive effects of SARS-CoV-2 infection in the lungs have brought ARDS to the forefront of medicine and biomedical research during the COVID-19 pandemic. Lung tissue specimens from patients with COVID-19-related ARDS demonstrate findings typical of ARDS from many causes [53], including diffuse alveolar damage (DAD) [54]—a pathological pattern characterized by airspace edema, hyaline membranes, and evidence of alveolar epithelial cell hyperplasia [55]. However, reports of SARS-CoV-2-infected lung tissue also indicate marked heterogeneity in the extent of alveolar epithelial infection at both macro- [56,57] and microscopic [58] levels. These findings likely reflect the known heterogeneity of inhaled particle deposition in the lungs [59] but may also result, at least in part, from variable expression of viral entry proteins across lung epithelial cell subpopulations [60] and developmental stages [61]. Nevertheless, widespread induction of cellular interferon responses extends to uninfected lung regions in COVID-19-related ARDS, demonstrating that virus-induced danger signals are transmitted beyond regional sites of injury [57]. The clinical relevance of the transmitted interferon responses is supported by recent genomic data that identify a strong association between interferon signaling genes and COVID-19 severity in large, diverse populations [62,63,64]. Interestingly, the “cytokine storm” found in COVID-19 ARDS may result from an interferon response that is, in fact, hypofunctional and fails to control the initial infection [63,65,66,67,68]. 

### 3.2. Direct Mechanisms of Alveolar Epithelial Damage

Mechanisms of lung injury that lead to ARDS center on alveolar epithelial cell damage, barrier dysfunction, and surfactant system impairment, resulting in widespread pulmonary edema formation (reviewed in [69]). Recent data reveal important roles for the alveolar epithelial glycocalyx in lung injury pathogenesis. The glycocalyx regulates cellular barrier function and cell adhesion (reviewed in [10]), and glycocalyx shedding can mediate lung disease [70,71,72,73]. In patients with ARDS, glycosaminoglycan accumulation in respiratory specimens, likely a marker of glycocalyx proteoglycan shedding, correlates with alveolar barrier permeability indices, duration of mechanical ventilation, and matrix metalloproteinase 7 (MMP-7) levels [72,73]. MMP-7, via shedding of syndecan-1, a proteoglycan prominently expressed by the lung epithelium, promotes alveolar airspace neutrophil recruitment and activation after various injuries [74,75]. However, syndecan-1 shedding may also promote a dysregulated inflammatory response by altering surfactant function and epithelial pro-survival signals, leading to lung injury [73,76]. Taken together, these findings support glycocalyx shedding as a new biomarker in ARDS and suggest that modulation of the shedding response may be therapeutic (Figure 1). 

Intra-alveolar hemorrhage is a long-recognized pathological feature of ARDS [77], and recent data suggest cell-free hemoglobin (CFH) may play an active role in driving lung injury [78]. Experimentally, CFH instillation into murine lungs induces alveolar barrier dysfunction and alveolar airspace inflammation [78], and alveolar permeability may be altered via the disruptive effects of CFH on AT1 cell bioenergetics [79]. The deleterious effects of CFH may not be restricted to epithelial surfaces, as endothelial exposure to CFH also induces fluid barrier hyperpermeability in cultured lung endothelial cells and isolated human lungs [80]. Together, these findings reveal that CFH can disrupt alveolar barrier function and promote ARDS development (Figure 1). 

Other recent findings build further on the growing mechanistic understanding of ARDS pathogenesis. Two reports indicate that influenza A virus (IAV) causes alveolar barrier dysfunction by disrupting alveolar epithelial junctional proteins [81,82]. Moreover, findings by Ruan et al. demonstrate IAV-induced activation of the transcription factor Gil1 mediates junctional protein loss in cultured alveolar epithelial-like cells and mouse models of IAV infection [82]. Following the onset of barrier hyperpermeability, loss of alveolar epithelial Na,K-ATPase, ENaC, and CFTR protein by IAV [83,84] and hypercapnia [85] may impair clearance of alveolar airspace fluid, potentiating pulmonary edema responses. In addition, lung injury may be induced or exacerbated by pathogen-induced alveolar epithelial responses that alter surfactant composition [86] and dysregulate alveolar epithelial cell metabolism, leading to airspace inflammation [87]. Although airspace inflammation results from many signaling mechanisms (reviewed in [88]), new data show pro-inflammatory responses to IAV lung infection are fine-tuned by the alveolar epithelial activity of the linear ubiquitin assembly complex (LUBAC)—a regulator of NFkB-dependent inflammation [89]. 

Chemical inhalation is an important but poorly understood cause of lung injury related to industrial accidents and military and terrorist activity. Recent data provide new insights into alveolar epithelial responses that lead to chemical lung injury. For example, mice exposed to ricin, a plant-derived exotoxin, demonstrate rapid ricin binding to lung epithelial cells, and AT2 cells may be particularly susceptible to ricin binding and ricin-induced cellular damage [90]. Similar to ricin, chlorine (Cl2) gas seems to cause major AT2 cell damage, as evidenced by alterations of lung surfactant protein content in Cl2-exposed mice [91]. Additional alveolar epithelial responses to Cl2 gas include loss of alveolar epithelial bioenergetics and loss of alveolar barrier function [92]. Rats exposed to hydrogen sulfide (H2S) also respond with increased alveolar barrier permeability, and studies using cultured alveolar epithelial-like cells suggest that H2S-induced barrier loss may be augmented by alveolar epithelial loss of ENaC expression [93]. Together, these new findings highlight alveolar epithelial damage, manifested in barrier and surfactant dysfunction, as a common response to chemical lung injury and suggest therapeutic approaches that target the alveolar epithelium may be beneficial.

Mechanical ventilation is a mainstay of clinical care for patients with ARDS and respiratory failure, but its application can induce pressure-related lung injury (reviewed by [94]). Although low tidal volume ventilation protects against ARDS-induced mortality [95], regional heterogeneity in alveolar edema patterns [96] generates persistent risk for alveolar exposure to injurious conditions, even with lung-protective ventilation strategies. New data show lung injury by ventilation-induced lung distension results from cellular mechanosensing pathways involving the alveolar epithelial nuclear membrane and mTOR complex 1 (mTORC1), leading to pulmonary edema, surfactant dysfunction, and lung stiffness [97,98]. The finding that ventilator-induced lung injury causes mTORC1 activation and surfactant dysfunction is particularly intriguing, since the mTORC1 activation occurs primarily in the small airway epithelium [98]. As the small airways are a major site of mechanical strain during high tidal volume ventilation [99] but not sources of surfactant dysfunction in ARDS [100], the relationship between airway-centered mTORC1 activation and surfactant abnormalities suggests stretch-induced signals in the small airway epithelium may be transmitted to the alveolar epithelium. Thus, these findings support the need for better understanding of the role of small airway responses in lung injury and their relationship to alveolar function.

### 3.3. Alveolar Epithelial Cell–Cell Communication Amplifies Injury Responses

IAV lung infection induces a robust cellular interferon response that limits viral infection but also promotes inflammation-induced lung injury (reviewed by [101,102]). New findings show IAV-induced interferon responses in infected alveolar epithelial cells are communicated to uninfected alveolar epithelial cells. Findings by Ramos et al. show cultured alveolar epithelial-like cells exposed to IAV express antiviral interferon-stimulated genes in a viral dose-dependent manner and induce interferon-stimulated gene expression in neighboring, uninfected cells [103]. 

To test the possibility that communication of interferon responses occurs in vivo, Stifter and colleagues generated a fluorescent reporter mouse that visualizes cellular interferon responses in IAV-infected lungs [104]. After intranasal IAV instillation, Stifter et al. found marked fluorescent protein expression in AT1 cells [104], indicating AT1 cells are uniquely responsive to IAV-induced interferon signaling. Since uninfected, fluorescent AT1 cells were located near infected AT2 cells [104], the findings suggest infected AT2 cells transmit signals to AT1 cells that activate AT1 cell interferon signaling. These new data support the notion that lung infection stimulates alveolar epithelial cell–cell communication in vivo and highlight a major role for AT1 cells in virus-induced interferon responses. Future research may clarify the significance of AT1 cell interferon responses for lung defense, inflammatory lung injury, and alveolar barrier function [105,106]. 

Alveolar epithelial cells are linked by gap junctional proteins that facilitate the intercellular transit of ions and small molecules, thus forming an extensive cell–cell communication network for the rapid coordination of cellular signals [107]. Alveolar epithelial gap junctional proteins were recently implicated in lung injury caused by *Staphylococcus aureus* (*S. aureus*) lung infection. Live imaging of intact mouse and human lungs indicate that contact with the alveolar epithelium causes inhaled *S. aureus* to form microaggregates in structural niches of the alveolar wall, where the bacteria stabilize, secrete the membrane pore-forming toxin alpha-hemolysin, and induce cytosolic Ca^2+^ increase in the adjacent alveolar epithelium [108]. Niche-localized Ca^2+^ signals spread to the epithelium of uninfected alveoli through connexin-43-containing gap junctional channels, leading to widespread loss of alveolar fluid barrier function and fatal lung injury [108]. These findings reveal major roles for alveolar microanatomy and epithelial cell–cell communication in lung injury pathogenesis, in that alveolar niches provide sites for bacterial stabilization and alveolar epithelial gap junctions amplify the bacteria-induced injury signals (Figure 1). 

### 3.4. Alveolar Epithelial Cells Transmit Injury Signals to Microvessels

Pro-inflammatory stimuli in alveolar airspaces induce paracrine communication from the alveolar epithelium to the adjacent microvascular endothelium [109]. In alveoli microinstilled with acid, epithelial–endothelial communication is mediated by epithelial release of diffusible reactive oxygen species (ROS) [110]. New findings generated by live imaging of intact lungs show that acid-induced ROS release from the alveolar epithelium causes mitochondrial depolarization and cytoskeletal destabilization in the epithelium-adjacent endothelium, leading to alveolar barrier dysfunction [111] (Figure 1). A surprising finding is that the depolarized mitochondria recover polarization within 30 min of alveolar contact with acid [111]. Since acid-induced membrane pore formation in the alveolar epithelium is similarly transient [110], taken together, these findings suggest alveoli have considerable capacity for recovery after acid-induced cellular damage. 

Pericytes—mesenchyme-derived stromal cells that directly contact microvascular endothelial cells [112]—are located in the alveolar interstitium near the alveolar epithelium in developing [113] and adult [114] lungs. This unique location between the epithelium and endothelium may position pericytes to transmit alveolar epithelial-derived injury signals to the endothelium and thereby play a major role in lung injury development. Indeed, recent studies in mice have identified that pericytes function as immune sensors that respond to alveolar epithelial damage by promoting inflammatory cell recruitment [115,116,117,118]. 

### 3.5. Alveolar Epithelial Cells Organize Signaling Networks with Innate Immunity

New research shows the alveolar epithelium engages in dynamic interactions with the innate immune system after lung injury. Work by Peteranderl and colleagues reveals a paracrine signaling network between the alveolar epithelium and AMs that contributes to pulmonary edema formation [83]. IAV-infected alveolar epithelial cells secrete interferon alpha, which leads to release of TNF-related apoptosis-inducing ligand (TRAIL) from AMs [83]. TRAIL signaling to uninfected alveolar epithelial cells causes kinase-dependent degradation of the epithelial basolateral Na,K-ATPase and failure of airspace edema absorption [64] (Figure 1). These novel findings demonstrate that effects of alveolar epithelial injury, in this case from IAV infection, extend beyond infected cells to disrupt the physiologic function of uninfected cells. 

A burgeoning literature suggests AT1 cells are primary regulators of alveolar immunologic activity [104,119,120]. Although the concept of AT1 cells as orchestrators of distal lung inflammation is teleologically intuitive given their extensive surface area [4,5,121], the overall understanding of AT1 cell function has lagged behind that of other cell types, and thus AT1 cells have been traditionally considered primarily in terms of their barrier and fluid balance functions [121]. Lin et al. [122] reported a new mechanism by which AT1 cells facilitate neutrophil migration from microvessels into alveolar airspaces, thereby promoting a core feature of ARDS pathogenesis [123]. The findings show mice with global deletion of epithelial membrane protein 2 (Emp2)—a protein highly expressed by AT1 cells—have reduced numbers of neutrophils in bronchoalveolar lavage fluid after intranasal instillation of bacteria or lipopolysaccharide (LPS) [122]. Epithelial Emp2 deletion blocks transepithelial neutrophil migration to cause an increase in neutrophils in the lung interstitium, particularly in the surrounding small airways [122]. The neutrophil migration defect is attributed to epithelial absence of Emp2-mediated expression of neutrophil adhesion molecules [122]. These findings shed new light on mechanisms by which neutrophils migrate into alveolar airspaces and implicate AT1 cells as central determinants of the migration. Further research is needed to clarify the extent to which alveolar versus airway Emp2 expression is responsible for interstitial neutrophil accumulation, as well as the potential role of Emp2 in alveolar epithelial barrier function and barrier recovery. 

In addition to promoting injurious lung inflammation, interactions between the alveolar epithelium and innate immune cells, most notably resident AMs, protect against lung injury. Recent studies show depletion of lung-resident AMs by CD11c-targeted Stat5 deletion augments LPS-induced lung inflammation and barrier dysfunction, likely through loss of AM suppression of epithelial cytokine secretion [124]. Genetic reduction of AMs augments signaling through alveolar epithelial arachidonic acid pathways to enhance the susceptibility of AT1 cells to IAV infection and increase IAV-induced lung injury and mortality [125], thus revealing a critical role for AMs in AT1 cell protection against inhaled pathogens. AMs directly interact with the alveolar epithelium via gap junctions to communicate anti-inflammatory cytosolic Ca^2+^ signals, limiting LPS-induced lung injury [126] (Figure 1). Interestingly, AM number and function become attenuated with age [127]. Considering the immunosuppressive and protective effects of AMs on the alveolar epithelium, age-related numeric and functional deficiencies in AMs likely contribute to the increased incidence of ARDS in older people. 

## 4. Alveolar Epithelial Repair and Regeneration 

### 4.1. Alveolar Epithelial Survival after Lung Injury

Recovery from lung injury requires that alveoli regain gas exchange function through the clearance of pulmonary edema fluid and inflammatory cells and reestablishment of the alveolar epithelial barrier. The extent to which barrier reestablishment depends on the regain of function in surviving alveolar epithelium versus regeneration of non-surviving alveolar epithelium is not clear. Although the prevailing view is that alveolar epithelial cell death is a major response to lung injury [128,129,130], recent studies suggest a considerable proportion of the injured alveolar epithelium, including AT1 cells, survive injury [49,119]. Using genetically modified IAV to identify AT1 and AT2 cells that survive direct infection, Fiege and colleagues report that alveolar epithelial cell survival correlates with time-dependent loss of surface antigen presentation, hence avoidance of CD8-T-cell-mediated cell killing [131]. Notably, survivor cells do not appear to be contiguous [131], suggesting that infected cells or cells with survival capability are unevenly distributed across the alveolar epithelium. Other mechanisms that may promote alveolar epithelial survival include pathogen-induced mitophagy [132] and AT2 cell secretion of leukemia inhibitory factor (LIF) [133,134]. 

### 4.2. Alveolar Epithelial Cell Contributions to Alveolar Epithelial Repair and Regeneration

**Figure 2:**
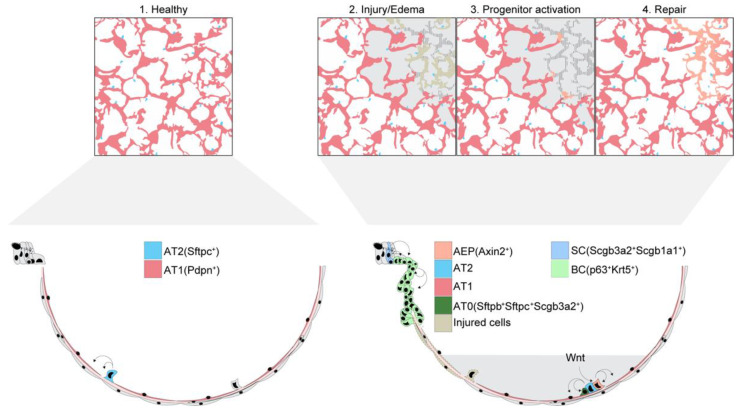
**Alveolar epithelial responses that determine alveolar repair and regeneration**. The schematic figures **1–4** show mechanisms of alveolar repair after lung injury. **(1)** In health, the alveolar epithelium is a continuous monolayer of squamous alveolar type 1 cells (AT1) and cuboidal alveolar type 2 (AT2) cells. **(2)** After severe lung injury, recovery of gas exchange function requires the replacement of damaged alveolar epithelial cells and the restoration of alveolar architecture. **(3)** Alveolar epithelial progenitors (AEPs) respond robustly to local production of Wnts to **(4)** rapidly expand and replenish AT2 cells. In turn, AT2 cells transition through the AT0 cell state to differentiate into AT1 cells. Other cells that may contribute to alveolar repair and regeneration include distal airway basal-like cells (BCs) and secretory cells (SCs).

A rapidly growing literature indicates that distal lung cell populations involved in alveolar epithelial repair and regeneration after lung injury may be more heterogeneous than traditionally considered (Figure 2). Although AT2 cells are established AT1 cell progenitors that respond to injury by proliferation and differentiation into AT1 cells [50,115,135], recent data show successful alveolar epithelial repair and regeneration may also incorporate contributions from AT1 cells and the distal airway epithelium. 

During development, AT1 cells may arise independently from a common alveolar epithelial progenitor [136] or from a subpopulation of AT2 cells [137]. However, after birth and in response to lung injury, AT2 cells are a major source of AT1 and AT2 cell renewal [48,49,136,138,139,140]. New findings show the regenerative capacity of AT2 cells may be determined by signaling by Wnt [48,141], hypoxia-inducible factor 1 alpha (HIF-1a) [138,139], Notch [140], and fibroblast growth factor 2 (Fgfr2) [142]. AT2 subpopulations with high regenerative capacity may be uniquely responsive to regenerative signaling. For example, a Wnt-responsive AT2 cell population characterized by Axin2 expression [48,141] is primed to enter the cell cycle and rapidly expand to regenerate alveolar epithelia after IAV-induced lung injury [141]. Bleomycin instillation induces a HIF-1a-responsive AT2 cell subset to proliferate and differentiate into AT1 cells [138], and a subpopulation of AT2 progenitor cells with low surfactant protein C (SFTPC) and high programmed death-ligand 1 (PD-L1) expression proliferate after pneumonectomy, perhaps through a mechanism involving Fgfr2 signaling [143]. 

In the process of repairing damaged alveoli, AT2 cells assume a transitional state before fully differentiating into AT1 cells. In human and primate lungs, AT2 cells transition to “AT0” cells, which may be bipotent in their ability to differentiate into AT1 or distal airway cells [144]. Lineage tracing studies in mice characterize similar transient alveolar epithelial cell subsets [145]. AT2 transdifferentiation may be regulated by growth factors [142] and cytokine signaling [138]. Interestingly, the inflammatory cytokine interleukin 1 beta (IL-1β) both supports transitional cell formation and arrests AT1 cell differentiation if signaling is prolonged [138]. These findings suggest inflammation may impede repair, thereby promoting persistent injury and creating a feed-forward loop of dysregulated inflammation characteristic of ARDS. 

Despite the traditional view that AT1 cells are terminally differentiated, recent studies suggest they retain plasticity and de-differentiate to give rise to alveolar epithelial cells [146,147]. However, the contribution of AT1 cells to alveolar epithelial regeneration after lung injury remains unclear, as the bulk of the evidence for AT1 cell plasticity in adult lungs was generated using pneumonectomy models and organoids [146,147]. Nevertheless, the possibility that AT1 cells retain the capacity to proliferate and differentiate into alveolar epithelial cells, perhaps during intermediate stages of AT1 cell homeostatic regeneration, as suggested by others [147], is intriguing. 

### 4.3. Distal Airway Cell Contributions to Alveolar Epithelial Repair and Regeneration

New data suggest the alveolar epithelium of injured lungs may be regenerated by progenitor cells from the distal airways. Using human lung tissue, Basil and colleagues reported the discovery of a secretory cell population marked by high levels of secretoglobin family 3A member 2 *(SCGB3A2*) and *SCGB1A1* gene expression and located in the distal-most portions of respiratory bronchioles, near alveoli [148]. Isolated cells from this population spontaneously decrease SCGB3A2 expression and develop AT2 cell characteristics, including lamellar bodies and SFTPC expression, when cultured in specific media [148]. In mice infected with IAV, distal airway basal-like cells activate a regeneration program marked by expression of the transcription factor p63 (p63) and keratin 5 (Krt5) and may migrate into damaged alveolar regions [149,150,151]. However, the extent to which p63/Krt5-expressing basal-like cells contribute to alveolar repair and regeneration is unclear. A population of club-like distal airway cells with high histocompatibility 2, K1 (H2-K1) expression is reported to have alveolar regenerative capacity and differentiate into AT1 and AT2 cells after bleomycin-induced lung injury [152]. Bronchioalveolar stem cells (BASCs)—specialized progenitor cells found at bronchioalveolar duct junctions—proliferate and contribute to alveolar epithelial regeneration after naphthalene injection [153]. Genetic lineage tracing shows the capacity of BASCs to reconstitute both airway and alveolar epithelium in bleomycin-induced lung injury models [154]. Although, taken together, these findings provide strong evidence that distal airway cells contribute to alveolar epithelial regeneration after lung injury, interpretations of the data derived from mouse models are limited due to the anatomical differences between the distal airways of mice and humans. As reviewed by Weibel [155] and recently illustrated by Basil and colleagues [148], human lung airways terminate in respiratory bronchioles and alveolar ducts and lack bronchioalveolar duct junctions. Thus, going forward it will be important to validate findings generated in mice in human lungs.

## 5. Maladaptive Alveolar Epithelial Responses That Cause Lung Remodeling

Most patients who survive ARDS experience complete recovery of lung function [156,157]. However, fibrosis, evidence of ineffective alveolar regeneration, and an altered immune landscape are common pathological features of severe ARDS, particularly in non-survivors [158,159,160,161,162]. Lungs of patients with severe ARDS due to COVID-19 show extensive evidence of lung injury and fibrosis [163,164], and recent findings show patients who survived H1N1 IAV-induced ARDS had persistent lung abnormalities visible on high-resolution computed tomography (CT) scans at three and six months post-ARDS [165]. Thus, in some patients, lung recovery from ARDS may be absent or prolonged. Dysregulation of the aforementioned repair mechanisms and subsequent aberrant lung remodeling may be drivers of the maladaptive remodeling that causes chronic lung dysfunction. Here, we review the recent literature to explore how dysregulated alveolar repair and regeneration drives maladaptive lung remodeling in patients with ARDS.

### 5.1. Evidence of Maladaptive Lung Remodeling in ARDS

Studies of human lung tissue support the notion that failure of alveolar repair and regeneration mechanisms underlie the chronic lung dysfunction that can result from ARDS. Analysis of lung tissues from ARDS patients shows evidence of delayed re-epithelialization of the alveolar epithelium, indicative of either reduced post-injury AT2 proliferation or defective AT2-to-AT1 cell transdifferentiation [161]. A report by Ting and colleagues identifies diffuse airspace edema, alveolar epithelial denudation, and abundant transitional cells in lung tissue from patients with COVID-19-related ARDS, suggesting that failed or prolonged AT2-to-AT1 cell transdifferentiation may explain persistent barrier permeability and respiratory failure [162]. 

Human lung tissue specimens also demonstrate infiltration of fibroproliferative immune populations in the distal parenchyma, particularly in patients with COVID-19. Lung tissue from patients infected with SARS-CoV-2 show accumulation of CD163-expressing monocyte-derived macrophages that express genes associated with fibrogenesis, including *SPP1*, *TGFB1*, *TGFBI*, *LGMN*, and *CCL18* [166]. In addition, peripheral blood mononuclear cell (PBMC)-derived monocytes exposed to SARS-CoV-2 are enriched in gene sets associated with fibrotic responses to a greater extent than monocytes exposed to IAV [166]. Importantly, CD163-expressing monocyte-derived macrophages may associate with fibroblasts in the lungs of patients with COVID-19-related ARDS [166] and are implicated in fibrosis progression in idiopathic pulmonary fibrosis (IPF) [167], suggesting CD163-expressing monocyte-derived macrophages may represent a link between ARDS and fibrotic lung disease. These data suggest the specific causes of alveolar injury and subsequent immunologic response are major determinants of failed lung recovery after ARDS.

### 5.2. Mechanisms of Post-ARDS Maladaptive Remodeling

**Figure 3:**
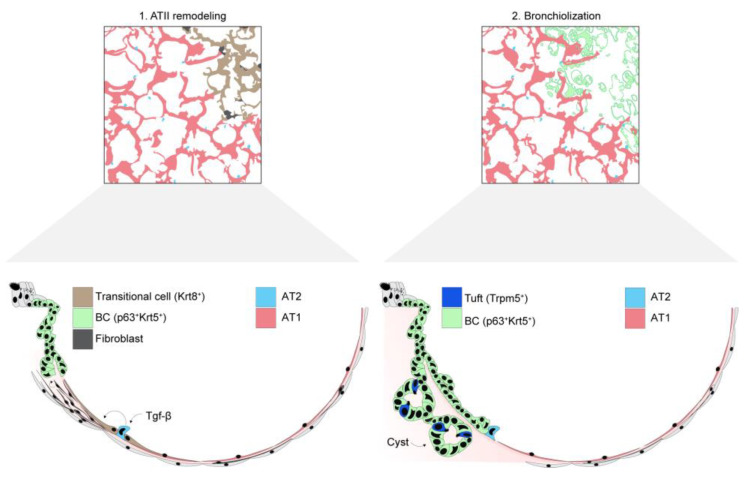
**Alveolar epithelial responses that determine maladaptive lung remodeling.** Schematic representations **1–2** represent proposed mechanisms of maladaptive lung remodeling after injury. **(1)** Inappropriately sustained TGFβ signaling blocks AT2-to-AT1 cell transdifferentiation, leading to accumulation of Krt8-expressing transitional cells. The presence of these cells is associated with the development of pulmonary fibrosis. **(2)** Basal-like cells (BCs) expand after injury to promote barrier recovery after extreme injuries. In the process, basal-like cell pods emerge at sites of damaged alveoli and form cystic structures. Tuft cells are found near BCs, but their functional significance is not yet clear.

AT2 cell progenitor dysfunction is a proposed mediator of post-ARDS maladaptive lung remodeling (Figure 3). After lung injury, proliferating AT2 cells enter a transitional state marked by loss of AT2 cell markers and increased cytokeratin expression [168,169,170]. In mouse models of recovery after bleomycin-induced lung injury, AT2 cells transiently acquire keratin 8 (Krt8) expression while transdifferentiating to AT1 cells [169]. However, studies of lung tissues from patients with IPF show the presence of Krt8/Krt18-expressing alveolar progenitors in areas of alveolar injury [169,170], suggesting persistence of the Krt8/Krt18-expressing transitional state may play a role in fibrosis development [170]. Since Krt8/Krt18-expressing transitional cells are overrepresented in both ARDS and end-stage fibrotic lung disease [162,169], accumulation of Krt8/Krt18-expressing transitional cells after lung injury could signal the presence of a maladaptive damage-and-repair cycle that inhibits AT2-to-AT1 transdifferentiation. 

Complete differentiation of Krt8/Krt18-expressing AT2 cells to AT1 cells is largely mediated by transforming growth factor beta (TGFβ) signaling [168,169,170,171]. To determine how TGFβ regulates AT2-to-AT1 cell transdifferentiation, Riemondy et al. carried out single cell RNA sequencing on AT2 cells obtained after LPS-induced lung injury in mice [168]. Transcriptionally distinct, injury-specific AT2 cell subpopulations emerged that included AT2 cells that were proliferating, in cell cycle arrest, and transdifferentiating [168]. AT2 cells in cell cycle arrest showed high expression of the TGFβ pathway genes *ITGB6*, *FN1*, *TGFB2*, *PDGFB*, *TGFBI*, and *TPM2* [168]. In cultured AT2 cells, TGFβ signaling induced cell cycle arrest, and TGFβ inhibition promoted AT2-to-AT1 transdifferentiation [168]. Together, these findings support an emerging paradigm in which TGFβ signaling halts the proliferation of AT2 cells after lung injury, then the withdrawal of TGFβ signaling induces AT2-to-AT1 cell transdifferentiation. As suggested by Riemondy and colleagues, persistent, inappropriate TGFβ signaling may therefore drive maladaptive lung remodeling in injured lungs. Future research may identify the upstream mechanisms and cellular sources responsible for TGFβ signaling after lung injury. 

Exogenous keratin-expressing cells accumulate at sites of alveolar damage in lung tissues of patients with ARDS [161,172] and may alter the course of alveolar repair. Mouse models demonstrate basal-like cells expressing p63/Krt5 migrate from the distal airway to injured alveoli after IAV lung infection but may not contribute to alveolar regeneration or the reestablishment of a gas exchange surface [149,150,151,173]. Rather, the cells persist for months and occasionally form cystic structures that express airway epithelial markers but no markers characteristic of the alveolar epithelium [173]. Similar findings have been recently reported in lungs from patients with ARDS, which demonstrate accumulation of p63/Krt5-expressing basal-like cells and peribronchiolar bronchiolar metaplasia (“bronchiolization”) in regions of alveolar damage [161,174]. Tuft cells emerge in areas of dysplastic alveoli along with the Krt5-expressing basal-like cells and may regulate inflammation after viral lung injury [175,176]. 

Although mouse lineage tracing studies indicate that p63/Krt5-expressing basal-like cells are derived primarily from airway epithelia [150,172,174,177,178], recent studies suggest human AT2 cells may also be a source of Krt5-expressing basal-like cell populations through metaplastic transdifferentiation [171]. Similar to Krt8/Krt18-expressing AT2-derived transitional cells [168], Krt5-expressing AT2-derived basal-like cells respond to TGFβ signaling [171], suggesting a potential lineage relationship between the two cell types. It remains to be seen whether basal-like cell populations found within damaged alveolar regions are basal fate-committed or regain alveolar plasticity at a later time post-injury. In addition, future research may increase understanding of the role of keratin-expressing basal-like cells in recovery from lung injury. Early expansion of basal-like cell populations may contribute to the re-establishment of the lung barrier, but proper clearance of the basal-like populations from damaged alveolar regions is likely an important step in the recovery of alveolar function.

Advanced age is a risk factor for ARDS-related mortality [179]. Mechanisms of increased age-related susceptibility to lung injury may relate to age-associated cellular senescence and its effects on alveolar repair, inflammation, and fibrogenesis. Multiple animal studies have assessed the role of cellular senescence in AT2 cell injury and repair [180,181,182,183]. Mice aged 72 weeks prior to inoculation with LPS show increased LPS-induced mortality and lung stiffness compared with young mice, and AT2 cells from LPS-exposed aged mice demonstrate decreased expression of cellular proliferative and apoptotic markers and increased expression of senescence and pro-inflammatory genes [181]. In addition, genetic models of AT2 cell senescence recapitulate findings seen in pulmonary fibrosis [183], and recent studies suggest roles for cellular senescence in the regulation of immune microenvironments [167,180,184]. Despite active investigation in this area, the role of AT2 cell senescence in the regulation of lung recovery and maladaptive remodeling remains unclear. Better understanding is needed of how senescence bears on the lung’s capacity for alveolar repair and regeneration, particularly in human lungs, as well as the therapeutic potential of senescence pathway targeting. 

## 6. Conclusions

This review of the recent literature highlights key advances in the understanding of the alveolar epithelium in relation to health, lung injury, and lung repair and regeneration. Recent findings have moved the field beyond the traditional AT1 and AT2 cell paradigm to reveal new cell subpopulations that maintain alveolar homeostasis, communicate injury signals, and participate in normal and maladaptive repair. Emerging data illuminate the complexity of alveolar physiology and pathology to provide a more complete picture of how alveoli maintain health and respond to injurious stimuli. 

Many of the recent advances were generated using increasingly sophisticated technologies, such as in vivo lung imaging techniques that allow for real-time assessment of host–pathogen interactions and single-cell transcriptomics that enable deep spatiotemporal exploration of cellular phenotypes. New methods continue to be identified that can resolve proteomes, metabolomes, and epigenomes in individual cells and are complemented by the development of biocomputational tools for analysis of the wealth of data generated by these high-throughput technologies. Thus, the framework provided here for the alveolar epithelial response to injury will continue to evolve with deeper understanding of the mechanisms of normal and abnormal repair and provide a springboard for future investigation, perhaps leading to the development of novel therapeutic approaches for ARDS. 

## Data Availability

Not applicable.
